# A painful pustular eruption after initiation of rifampin, isoniazid, pyrazinamide and ethambutol therapy

**DOI:** 10.1016/j.jdcr.2025.06.017

**Published:** 2025-06-21

**Authors:** Lauren Fleshner, Mehmet Fatih Atak, Austin Jabbour, Kenneth Shulman, Banu Farabi, Shoshana Marmon

**Affiliations:** a School of Medicine, New York Medical College, Valhalla, New York; b Dermatology Department, NYC Health + Hospital/Metropolitan, New York, New York; c Dermatology Department, NYC Health + Hospitals/South Brooklyn Health, Brooklyn, New York

**Keywords:** cutaneous tuberculosis, papulonecrotic tuberculid, tuberculosis

## Case presentation

**Fig 1:**
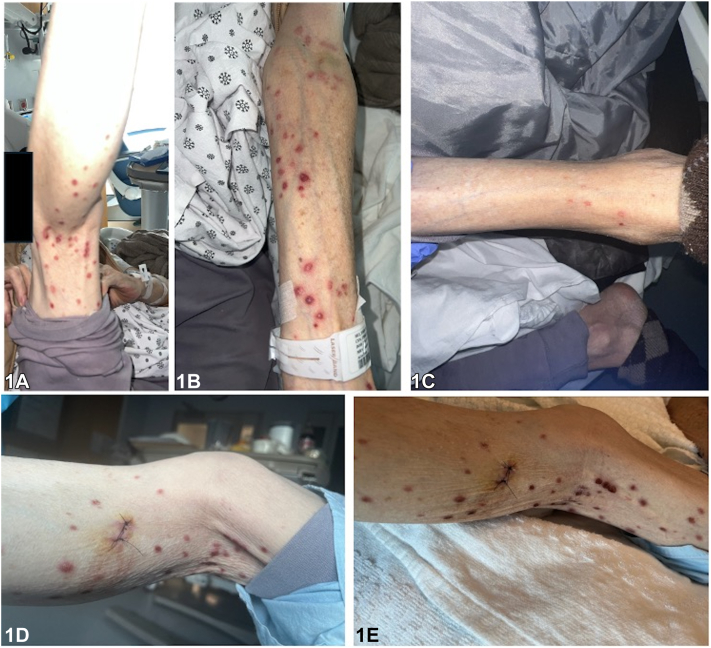

**Fig 2:**
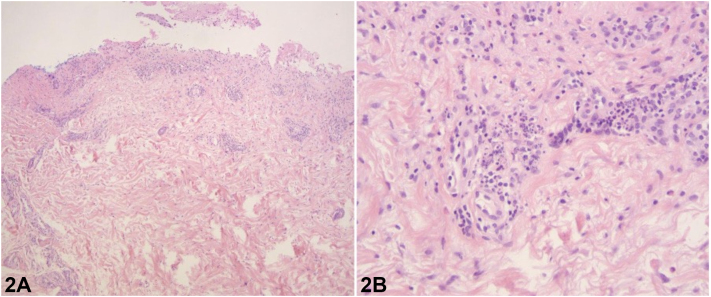

A 69-year-old male with active pulmonary tuberculosis and a history of IgA nephropathy presented with a generalized pruritic and painful eruption that developed within days of initiating rifampin, isoniazid, pyrazinamide and ethambutol (RIPE) therapy. RIPE treatment was held, and dermatology was consulted. The patient reported subjective fever, productive cough, and significant weight loss, but denied any previous episodes of similar rash, underlying dermatological conditions, or recent exposure to hot water through recreational activity. Physical examination revealed tender, erythematous papules, papulonodules and pustules scattered across his extremities, abdomen, and back (Fig 1, *A*-*D*). Several lesions had developed central necrotic crusts (Fig 1, *E*). No mucosal lesions were noted. A punch biopsy of the left lower extremity was performed. Histopathologic examination showed vesicular dermatitis with prominent neutrophilic and eosinophilic infiltration, accompanied by vasculitic changes (Fig 2, *A* and *B*). Bacterial, mycobacterial, and viral cultures were negative.

**Question 1: Which of the following is the most likely diagnosis?**

A. Erythema induratum
B. Dermatitis herpetiformis (DH)
C. Lichen scrofulosorum (LS)
D. Papulonecrotic tuberculid
E. Acute generalized exanthematous pustulosis (AGEP)

**Answer:**

A. Erythema induratum – Incorrect. Erythema induratum are painful, violaceous nodules and plaques and are typically found in females on the posterior lower extremities. It is usually associated with tuberculosis. On histopathology, granulomatous inflammation and lobular panniculitis are characteristic.
B. Dermatitis herpetiformis (DH) – Incorrect. Although DH is associated with IgA nephropathy and may present with erythematous papules and pustules, the lesion distribution outside typical predilection sites and the histopathologic findings—lacking subepidermal vesicles and characteristic neutrophilic microabscesses—were not consistent with DH.
C. Lichen scrofulosorum (LS) – Incorrect. Although LS is a cutaneous manifestation of tuberculosis, it is primarily seen in children and typically presents as a lichenoid eruption with asymptomatic, skin-colored papules, which is not observed in this case.
D. Papulonecrotic tuberculid – Correct. Papulonecrotic tuberculids represent a type III hypersensitivity reaction to tuberculosis. They are typically characterized by a positive Mantoux test, evidence of current or prior TB infection, and clinical improvement with antituberculosis therapy.
E. Acute generalized exanthematous pustulosis (AGEP) – Incorrect. Although the rash appeared within days of starting RIPE therapy, the presence of papules and papulonodules is atypical for AGEP, which characteristically presents with numerous pin-sized, nonfollicular pustules on a background of diffuse erythema. Additionally, the vasculitic changes seen in histopathology are not commonly associated with AGEP.

**Question 2: Which of the following histopathologic features is *NOT* associated with papulonecrotic tuberculids?**

A. Numerous acid-fast bacilli
B. *Mycobacterium tuberculosis* DNA on polymerase chain reaction of skin lesions
C. Leukocytoclastic vasculitis
D. Granulomas and multinucleated giant cells
E. Wedge-shaped area of necrosis

**Answer:**

A. Numerous acid-fast bacilli – Correct. Papulonecrotic tuberculids are paucibacillary (very few or no organisms seen). Acid-fast bacilli are typically not identified in skin biopsies.^1^
B-E. Incorrect – Polymerase chain reaction may detect *M. tuberculosis* DNA in some skin lesions, despite the absence of visible organisms.^2^ Histopathologic features such as leukocytoclastic vasculitis, granulomas with multinucleated giant cells, and wedge-shaped necrosis have all been reported in papulonecrotic tuberculids.^3^^,^^4^

**Question 3: What is the next best step in management?**

A. Initiate systemic corticosteroids
B. Discontinue all treatment permanently
C. Repeat skin biopsy and cultures for acid-fast bacilli
D. Resume therapy
E. Begin antiviral therapy

**Answer:**

A. Initiate systemic corticosteroids – Incorrect. Systemic corticosteroids are immunosuppressive and may exacerbate tuberculosis, making them inappropriate in this setting.
B. Discontinue all treatment permanently – Incorrect. Permanent discontinuation of RIPE therapy could worsen the underlying infection. Papulonecrotic tuberculids generally respond well to continued anti-TB treatment.^4^^,^^5^
C. Repeat skin biopsy and cultures for acid-fast bacilli – Incorrect. As tuberculids represent a hypersensitivity reaction to TB elsewhere in the body, organisms are typically absent in the lesions, making repeat biopsy and cultures unnecessary.
D. Resume therapy – Correct. Papulonecrotic tuberculid typically responds rapidly to RIPE therapy.^4^^,^^5^ In this case, resuming RIPE therapy under infectious disease guidance led to complete resolution of the skin lesions.
E. Begin antiviral therapy – Incorrect. There is no clinical or histopathologic evidence of viral infection, and antiviral therapy is not indicated.

## Conflicts of interest

None disclosed.
